# Transcription of Interleukin-8: How Altered Regulation Can Affect Cystic Fibrosis Lung Disease

**DOI:** 10.3390/biom5031386

**Published:** 2015-07-01

**Authors:** Karim Jundi, Catherine M. Greene

**Affiliations:** Department of Medicine, Respiratory Research Division, Royal College of Surgeons in Ireland Education and Research Centre, Beaumont Hospital, Dublin 9, Ireland; E-Mail: karimjundi@rcsi.ie

**Keywords:** interleukin-8, cystic fibrosis, lung

## Abstract

Interleukin-8 (IL-8) is a neutrophil chemokine that is encoded on the *CXCL8* gene. Normally *CXCL8* expression is repressed due to histone deacetylation, octamer-1 binding to the promoter and the inhibitory effect of nuclear factor-κB repressing factor (NRF). However, in response to a suitable stimulus, the human *CXCL8* gene undergoes transcription due to its inducible promoter that is regulated by the transcription factors nuclear factor-κB (NF-κB), activating protein (AP-1), CAAT/enhancer-binding protein β (C/EBPβ, also known as NF-IL-6), C/EBP homologous protein (CHOP) and cAMP response element binding protein (CREB). *CXCL8* mRNA is then stabilised by the activity of p38 mitogen-activated protein kinase (p38 MAPK). Cystic fibrosis (CF) lung disease is characterised by a neutrophil-dominated airway inflammatory response. A major factor contributing to the large number of neutrophils is the higher than normal levels of IL-8 that are present within the CF lung. Infection and inflammation, together with intrinsic alterations in CF airway cells are responsible for the abnormally high intrapulmonary levels of IL-8. Strategies to inhibit aberrantly high *CXCL8* expression hold therapeutic potential for CF lung disease.

## 1. Introduction

Inflammation is an important consequence of tissue injury that can result from several causes such as infection, trauma, neoplasm, and autoimmune disease. This mechanism is highly regulated by small molecules called chemokines, which control trafficking of various types of leukocytes via interaction with transmembrane receptors. One important chemokine that plays a vital role in most inflammatory pathways is interleukin (IL)-8.

Based on its functions, in the past, IL-8 has been alternatively named as T-cell chemotactic factor, neutrophil-activity peptide 1, Beta-thromboglobulin-like protein, and tumour necrosis factor-induced gene, amongst others. However, the name and symbol chemokine (C-X-C motif) ligand 8 and CXCL8, respectively, were adopted and have been officially endorsed by the Chemokine Nomenclature Subcommittee of the International Union of Immunological Societies [1]; nonetheless the name interleukin-8 is still widely used when referring to the protein.

In humans, IL-8 is one of fifteen members of the CXC chemokine family that are mostly encoded on chromosome 4q, spanning a region of approximately 2.5 Mb [2]. The IL-8 gene (*CXCL8*) has been mapped to 4q12-q21 by using somatic cell hybridization and *in situ* hybridization. *CXCL8* is 3211 bases in length and is encoded on 4 exons. Monocytes/macrophages, epithelial cells, smooth muscle cells and endothelial cells can all produce IL-8. It exists in two isoforms; a 72 amino acid form derived from endothelial cells and the more abundant 77 residue form secreted by monocytes and other cells [3,4]. The main receptors that interact with IL-8 are G-protein coupled seven transmembrane receptors CXCR1 and CXCR2, where the former has greater affinity and expression [5,6].

Due to the significant role IL-8 has in the inflammatory process in various diseases, importantly its expression can be regulated through a number of mechanisms. For example A/U rich elements and microRNA recognition elements in the 3' untranslated region of the *CXCL8* mRNA have been reported to contribute to its post-transcriptional regulation [7,8]. However, of relevance here is the fact that the *CXCL8* promoter is regulated by a selection of inducible transcription factors.

A major property of IL-8 during the inflammatory process is chemotaxis of target cells to the site of inflammation, in particular neutrophils. IL-8 also has chemotactic activity against T cells and basophils. Neutrophil adhesion to and transmigration across the endothelium are regulated by IL-8 and once neutrophils arrive to the site of inflammation, IL-8 further stimulates those cells to carry out phagocytosis, thus increasing the efficiency of tissue repair. Studies have also shown that IL-8 also has other immunomodulatory effects including the ability to induce matrix metalloproteinase-9 expression, release of TNF-related apoptosis-inducing ligand (TRAIL) and prime respiratory burst in neutrophils (reviewed in [9]).

## 2. Transcriptional Regulation of *CXCL8*

Inflammation recruits neutrophils to the site of injury, by a mechanism that is directed by chemokines. In the cystic fibrosis (CF) lung the most abundant chemokine is IL-8; it has variable characteristics, of which the most remarkable is its inducible expression which allows for variation of its expression levels. In non-pathological tissue, levels of IL-8 are not detectable, but are exponentially increased by 10- to 100-fold in response to pro-inflammatory cytokines such as tumour necrosis factor (TNF) and IL-1, bacterial or viral virulence factors, and oxidative stress. The expression of IL-8 is highly coordinated by many complex integrated signalling pathways [10] and is stimulus-dependent as shown, for example, by Kasahara *et al.* who demonstrated this phenomenon through nuclear run-on experiments in astrocytes [11].

The proximal region of the *CXCL8* promoter spans approximately 200 nucleotides of the 5' flanking region of the *CXCL8* gene, and is essential for transcriptional regulation of that gene [12]. Three of the major mechanisms responsible for *CXCL8* regulation are: (i) repression of the *CXCL8* promoter; (ii) transcriptional activation by inducible transcription factors; and (iii) mRNA stabilization. The following sections discuss each of these in more depth.

### 2.1. Repression of the CXCL8 Promoter

*CXCL8* transcription is effectively repressed in unstimulated cells by a combination of three mechanisms: Deacetylation of histones, octamer-1 (Oct-1) binding, and active repression by NF-κB repressing factor (NRF). Gene transcription is normally enhanced by histone acetylation which improves the activity of transcriptional enhanceosomes (TE). TEs provide multi-protein surfaces that make optimal contact with the proteins of the basal transcriptional machinery and thus facilitate maximal gene transcription; deacetylation has the opposite effect. Histone deacetylase-1 (HDAC-1) inhibition derepresses expression of *CXCL8*, and following the recruitment of CREB binding protein (CBP)/p300 to the promoter results in hyperacetylation of histones and chromatin remodelling ultimately relieving the repression [13,14]. Regarding Oct-1, it has been shown that replacing the Oct-1 repressor with nuclear factor-κB (NF-κB) and CAAT/enhancer-binding protein (C/EBP) as a consequence of IL-1 stimulation induces transcription at the *CXCL8* promoter [15]. Likewise binding of NRF to a negative regulatory element (NRE) in the *CXCL8* promoter, which incompletely overlaps with the NF-κB response element, also represses *CXCL8* expression [16]. Experiments have shown that reduced cellular NRF levels, achieved by expressing anti-sense RNA, causes spontaneous *CXCL8* gene expression. Mutations in the NRE site result in loss of NRF binding and an increase in basal *CXCL8* transcription. Unusually, NRF has a dual role in *CXCL8* transcription: it acts as a repressor in the absence of a stimulus but actually functions as a co-activator to enhance IL-1-induced IL-8 protein production.

### 2.2. Transcriptional Activation of CXCL8 by Inducible Transcription Factors

**Figure 1:**
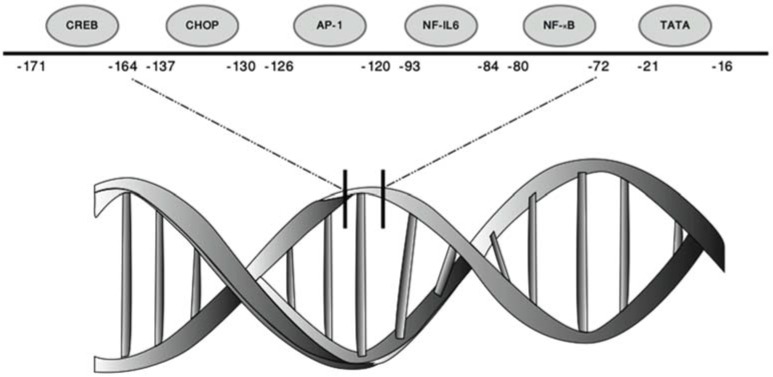
Architecture of the interleukin-8 promoter. Graphic representation of the *CXCL8* promoter showing the locations of the binding sites for transcription factors that induce its expression (adapted from reference [12]).

Through mutation and deletion analysis, it was discovered that the *CXCL8* promoter element contains NF-κB, activating protein (AP-1), and C/EBPβ (also known as NF-IL-6) binding sites [10]. In addition the transcription factors C/EBP homologous protein (CHOP) [17] and cAMP response element binding protein (CREB) [18] can also bind the *CXCL8* promoter (Figure 1 and Table 1).

NF-κB is a dimeric transcription factor composed of various homo- or heterodimeric combinations of 5 subunits: RelA (also called p65), RelB, c-Rel, p50 (generated from the precursor p105/NF-κB1) and p52 (generated from p100/NF-κB2). It is stored in the cytoplasm in its inactivated form by binding to its inhibitory proteins, IκBα and β. During a stress stimulus, NF-κB is activated by the phosphorylation of IκB proteins by the IκB kinases, IKKα/β and IKKγ/Nemo. This results in the ubiquitination and rapid degradation of the inhibitory proteins by the proteosome, allowing NF-κB to translocate to the nucleus and bind to the endogenous *CXCL8* promoter by its RelA subunit [19]. NF-κB is essential for activation of *CXCL8* transcription, while AP-1 and C/EBP are required for maximal *CXCL8* expression by synergising with NF-κB [10,20].

AP-1 is a homodimer or heterodimer composed of c-Jun, Jun-B, Jun-D, Atf-2, c-Fos, Fra-1, and Fra-2 [21]. In contrast to NF-κB which normally resides in the cytosol but translocates to the nucleus when IκB is released, AP-1 is directly bound to its target sequence on DNA and its trans-activating activity is regulated by its abundance, phosphorylation of transactivation domains, and the binding of protein kinases [22]. AP-1 is activated through MAPKs. Interestingly all three MAPK pathways have been shown to contribute to *CXCL8* gene expression: the extracellular-regulated protein kinase (ERK 1 and ERK 2), JUN-N terminal protein kinase (JNK), and p38 MAPK pathways [10].

The ERK pathway on its own is not a very potent inducer of IL-8, but has the potential to enhance IL-8 induction stimulated by NF-κB [23]. Regarding JNK signalling, Krause *et al.* showed how JNK similar to NF-κB, is essential for *CXCL8* expression [24], most likely via activation of c-Jun. A study by Holtmann *et al.* suggested that NF-κB and JNK work together for the induction of IL-8, as selectively blocking either pathway remarkably reduced IL-8 secretion [23]. p38 MAPK contributes to stabilisation of the *CXCL8* transcript (see below).

C/EBPs and CHOP are basic leucine zipper transcription factors; C/EBP-β (also named NF-IL-6) not only regulates expression of interleukin-6 but also can bind to the *CXCL8* promoter and co-operate with NF-κB for transcription of *CXCL8* [25]. CHOP regulates *CXCL8* expression independently of NF-κB [26]. Finally although CREB synergises with NF-κB to induce expression of interleukin-6, there is no synergy between these transcription factors in the process of *CXCL8* expression induced by TNFα in astrocytes [27].

### 2.3. CXCL8 mRNA Stabilization

Stabilization of the *CXCL8* mRNA is mediated by p38 MAPK. Both MKK6 and MK2 are also involved in this process. MKK6 is a dual specificity MAPK kinase that activates p38 MAPK, and MK2 (MAPK-activated protein kinase-2) is a downstream substrate of MKK6-p38 MAPK signalling [23,28,29]. Collectively this signalling pathway targets the A/U-rich 3' UTR of *CXCL8* mRNA and stabilizes the transcript.

**Table 1:** Characteristics of transcription factors that regulate *CXCL8* expression.

Transcription Factors	Transcription Factor Consensus Sequence	Subunits/Isoforms	Structure	Position bp: Base Pair
NF-κB	GGAATTTCC	NF-κB1 (p50 & p105)	Dimeric	−80/−72 bp
		NF-κB2 (p52 & p100)		
		C-Rel		
		RelA (p65)		
		RelB		
NF-IL6/C/EBPβ	AGTTGCAAAT	3 Isoforms:	Binds DNA as a dimer	−93/−84 bp
		P17676-1	Forms stable heterodimers with CEBPA, CEBPD and CEBPG	
		P17676-2		
		P17676-3		
AP-1	TGACTCA	c-Jun	Homodimer/heterodimer	−126/−120 bp
		JunD		
		JunB		
		Atf-2		
		c-Fos		
		Fra-1		
		Fra-2		
CHOP	GTGTGATG	Isoforms:	Heterodimer	−137/−130 bp
		P35638-1		
		P35638-2		
CREB	TTTCGTCA	Isoforms:	Binds DNA as a dimer	−171/−164 bp
		P16220-1		
		P16220-2		
		P16220-3		

## 3. Interleukin-8 in the Cystic Fibrosis Lung

**Figure 2:**
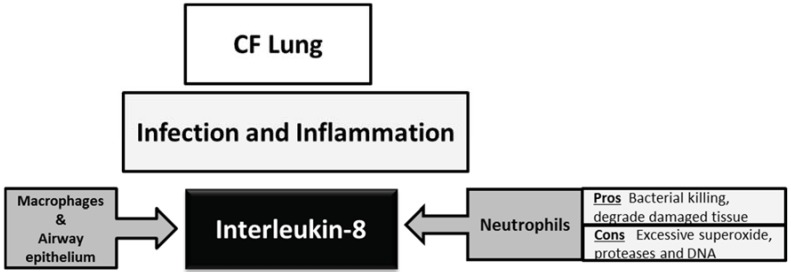
Effects of Interleukin-8 in the cystic fibrosis lung.

In cystic fibrosis defective or dysfunctional CFTR (cystic fibrosis transmembrane conductance regulator) impairs chloride secretion from airway epithelial cells, leading to excessive reabsorption of sodium and water, causing a reduction of airway surface liquid volume and an impairment of mucociliary clearance [30]. This predisposes to bacterial colonisation of the lung and is associated with neutrophil infiltration and inflammation. The purpose of neutrophil infiltration into the lung is to kill bacteria and degrade damaged tissues. However an over exaggerated influx of neutrophils can lead to excessive release of neutrophil granule contents including superoxide anions and enzymes such as neutrophil elastase (NE), causing damage to the epithelium and injuring the airway structure. Many studies have reported higher than normal levels of IL-8 in the CF lung and this is in large part responsible for the high neutrophil burden within the CF lung [31,32,33,34] (Figure 2).

IL-8 is a neutrophil chemokine produced principally by macrophages and epithelial cells in the CF lung in response to infective and inflammatory stimuli. Ideally this leads to neutrophil-mediated bacterial killing via CXCR1 and removal and degradation of damaged tissue [35]. Due to exaggerated number of neutrophils in the CF lung, there are higher than normal levels of superoxide, neutrophil proteases and DNA [36,37,38,39].

*Pseudomonas aeruginosa* is the most common CF lung pathogen. However many other microbes can also colonise the CF airways including *Staphylococcus aureus*, *Haemophilus influenzae*, *Burkholderia cepacia*, *Aspergillus fumigatus* and other fungi, as well as various anaerobic bacterial species [36]. People with CF are also prone to viral lung infections. Activation of toll-like receptors (TLRs) on epithelial or innate immune cells in the lung by microbial- and host-derived factors, can induce proinflammatory gene expression and in particular *CXCL8* expression. A number of studies have reported the expression and function of TLRs in the CF lung and in *in vitro* models of CF. mRNA for TLRs 1-10 is expressed by non-CF tracheal, bronchial and alveolar type II airway epithelial cells. Likewise all TLRs have been detected in primary and transformed CF airway epithelial cells [40,41,42]. TLR5, for example, is expressed by airway epithelial cells and can mediate inflammatory responses to flagellin-expressing *Pseudomonas aeruginosa*, *Burkholderia cepacia* and *B. cenocepacia* [43,44]. It has been shown that CF airway epithelial cells respond to *P. aeruginosa*, IL-1β or TNFα stimulation with an exaggerated NF-κB activation thereby strongly inducing *CXCL8* transcription [45,46,47]. This may be related to NADPH oxidase which is necessary for IL-8 synthesis in response to TLR activation by *P. aeruginosa* [48], however the response does not involve increased C/EBP [49]. Taken together these studies underscore the importance of infection and inflammation in contributing to the exaggerated expression of *CXCL8* in the CF lung.

A cycle of inflammation exists in the CF lung whereby neutrophil-derived products can drive *CXCL8* expression and IL-8 protein production in airway epithelial cells leading to further neutrophil recruitment. A major factor responsible for this is NE, a serine protease released from activated neutrophils. Its normal function is to degrade elastin however it can also affect proinflammatory gene expression, including *CXCL8*, in airway epithelial cells. The McElvaney group have reported the mechanism by which NE can induce IL-8 protein production from bronchial epithelial cells [50,51,52]. They demonstrated that NE cleaves meprin-α on the epithelial cell surface leading to trans-activation of the epidermal growth factor receptor (EGFR) by release of a specific EGFR ligand, transforming growth factor-α. EGFR then co-localises with TLR4 and an intracellular signalling cascade leading to NF-κB activation and *CXCL8* expression is implemented. This group also reported that NE can release heme from haemoglobin, possibly present in the CF lung due to micro bleeds, and that this in turn signals via a meprin/TLR/NF-κB pathway also leading to *CXCL8* induction [53]. NE has other deleterious effects in the CF unrelated to IL-8, including up regulation of mucin expression and the ability to degrade immunoglobulins, antiproteases, chemokine receptors and innate immunity proteins [35,54,55,56].

Various intrinsic defects in CF cells have been reported that contribute to aberrant *CXCL8* expression. For example, CF nasal airway epithelial cells have higher basal NF-κB activity and IL-8 protein expression compared to non-CF cells, however this effect is not evident in nasal epithelial cells stimulated with inflammatory or infective stimuli [57]. In CF airway epithelial cells Bartling and Drumm reported how defective CFTR contributes to oxidative stress resulting in intrinsic alterations in HDACs and increased acetylation of the *CXCL8* promoter [58]. Finally it has been shown that stabilisation of *CXCL8* mRNA in CF airway epithelial cells occurs due to constitutive p38 and ERK1/2 MAPK signalling [59].

## 4. Strategies Targeting CXCL8 in Cystic Fibrosis

Given the higher than normal levels of IL-8 in the CF lung and the deleterious consequences associated with this, the question arises as to whether IL-8 can be targeted therapeutically for CF? A number of groups have tested a variety of approaches including the use of anti-inflammatories, specific NF-κB inhibitors, transcription factor decoys and more recently microRNA-modulation approaches.

For example, in the CF lung CXCL8 is bound by gycosaminoglycans (GAGs); this does not interfere with its chemotactic activity and protects it from degradation by neutrophil proteases [60]. Treatment of people with CF with nebulized hypertonic saline can disrupt the interaction between IL-8 and GAGs rendering IL-8 susceptible to proteolytic degradation thereby decreasing neutrophil chemotaxis and facilitating resolution of inflammation [61]. More recent *in vitro* studies have shown that a CXCL8-based decoy, PA401, which displays no chemotactic activity but has GAG binding affinity can disrupt CXCL8:GAG complexes, rendering CXCL8 susceptible to proteolytic degradation [62]. CXCL8 normally promotes bacterial killing by neutrophils through CXCR1. However unopposed proteolytic activity in the CF airways cleaves CXCR1 on neutrophils and disables their bacterial-killing capacity. Hartl *et al.* demonstrated *in vivo* inhibition of proteases by inhalation of alpha1-antitrypsin leading to restored CXCR1 expression and improve bacterial killing in people with cystic fibrosis [35].

Regarding anti-inflammatory approaches, *in vitro* studies have tested the effects of genistein, fluvastatin and corilagin, amongst others [63,64,65]. Collectively these studies demonstrated that genistein reduces IL-8 production in cultured CF bronchial gland cells by increasing cytosolic IκBα protein levels, fluvastatin decreased IL-8 production in whole blood in response to *Pseudomonas* or *Aspergillus* antigens, and corilagin binds to NF-κB, thus inhibiting NF-κB/DNA interactions and can decrease* CXCL8* gene expression in CF bronchial IB3-1 cells.

The transcription factor decoy approach is based on molecules mimicking the target sites of transcription factors and interfering with their activity when delivered into target cells. Intracellular delivery of double-stranded oligodeoxynucleotides (ODNs) designed to block NF-κB binding to the *CXCL8* promoter in IB3-1 and CuFi-1 cells could partially inhibit the *P. aeruginosa*-dependent *CXCL8* transcription [66]. Similar ODNs delivered *in vitro* to IB3-1 cells significantly inhibited *P. aeruginosa* LPS-induced *CXCL8* mRNA expression [67]. Peptide nucleic acids (PNAs), in particular PNA-DNA-PNA (PDP) chimeras, are more stable than ODNs molecules. Finotti *et al.* have demonstrated that an NF-κB-targeting PDP chimera acts as a strong inhibitor of *CXCL8* gene expression [68].

Regarding microRNA modulation approaches to decrease CXCL8 production in the CF lung, three recent studies have determined how miRNAs that are aberrantly expressed in the CF airways and that regulate expression of *CXCL8,* may be targeted for therapeutic benefit [69,70,71]. The first reported on miR-155, a miRNA that is highly expressed in CF lung, regulates SHIP1 and indirectly leads to increased IL-8 production. An inhibitor of miR-155 (antagomir-155) reversed this effect *in vitro* [69]. The other two studies on this topic have investigated miRNAs that are decreased in the CF lung that directly target *CXCL8* mRNA. Fabbri *et al.* identified miR-93 as a miRNA that is decreased in IB3-1 and Cufi-1 cells infected with *P.* *aeruginosa.* Artificially increasing the levels of miR-93 (with pre-miR-93 transfection) could decrease IL-8 expression in these CF airway epithelial cells *in vitro* [70]. Lastly, Oglesby *et al.* [71] measured the expression and function of miRNAs decreased in the CF lung and in βENaC-transgenic mice that are predicted to target IL-8 mRNA. miR-17 was identified as a miRNA that regulates IL-8 and its expression was decreased in adult CF bronchial brushings, βENaC-transgenic mice and bronchial epithelial cells chronically stimulated with *Pseudomonas*-conditioned medium. Overexpression of miR-17 inhibited IL-8 protein production in F508del-CFTR homozygous bronchial epithelial cells. Thus modulating the expression of miRNAs that target *CXCL8* mRNA in CF bronchial epithelial is likely to represent a new therapeutic strategy for CF [71].

## 5. Conclusions

Much is known about the transcriptional regulation of *CXCL8* [12]. Detailed studies have identified the inducible regulatory factors involved and how these respond to different infective and pro-inflammatory stimuli. In the context of CF it is also known that intrinsic alterations due to defective CFTR can affect *CXCL8* transcription and mRNA stabilization. Various studies have demonstrated the feasibility of inhibiting *CXCL8* expression in CF airway epithelial cells and it is hoped that in the near future some of these strategies will be developed further and can be used for therapeutic benefit in individuals with CF.
